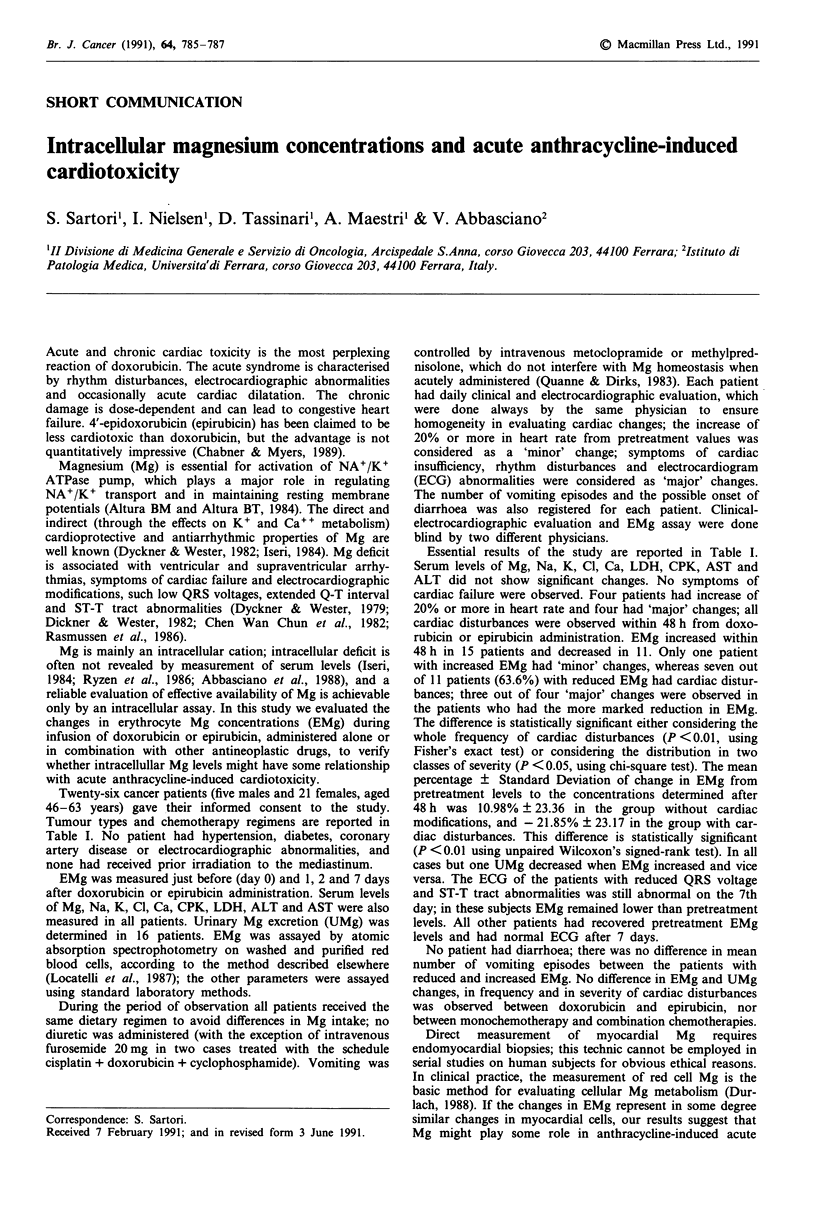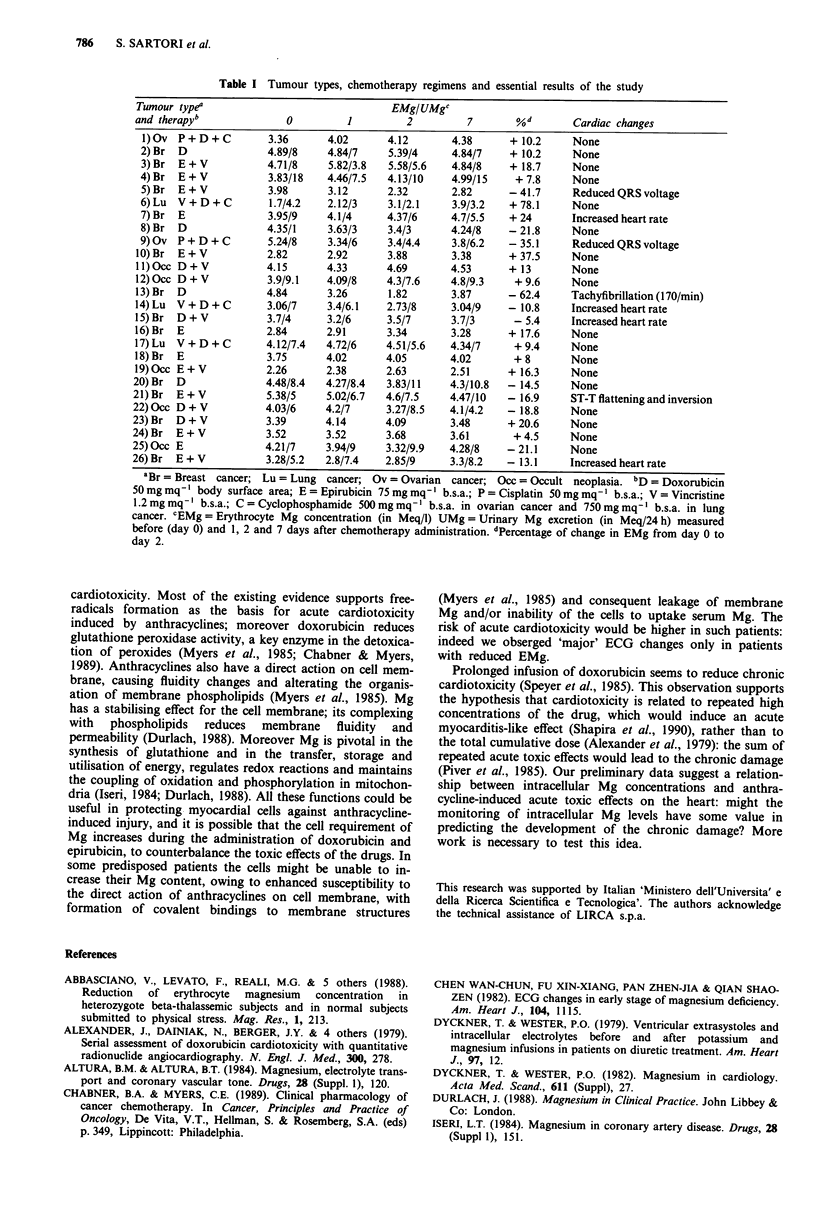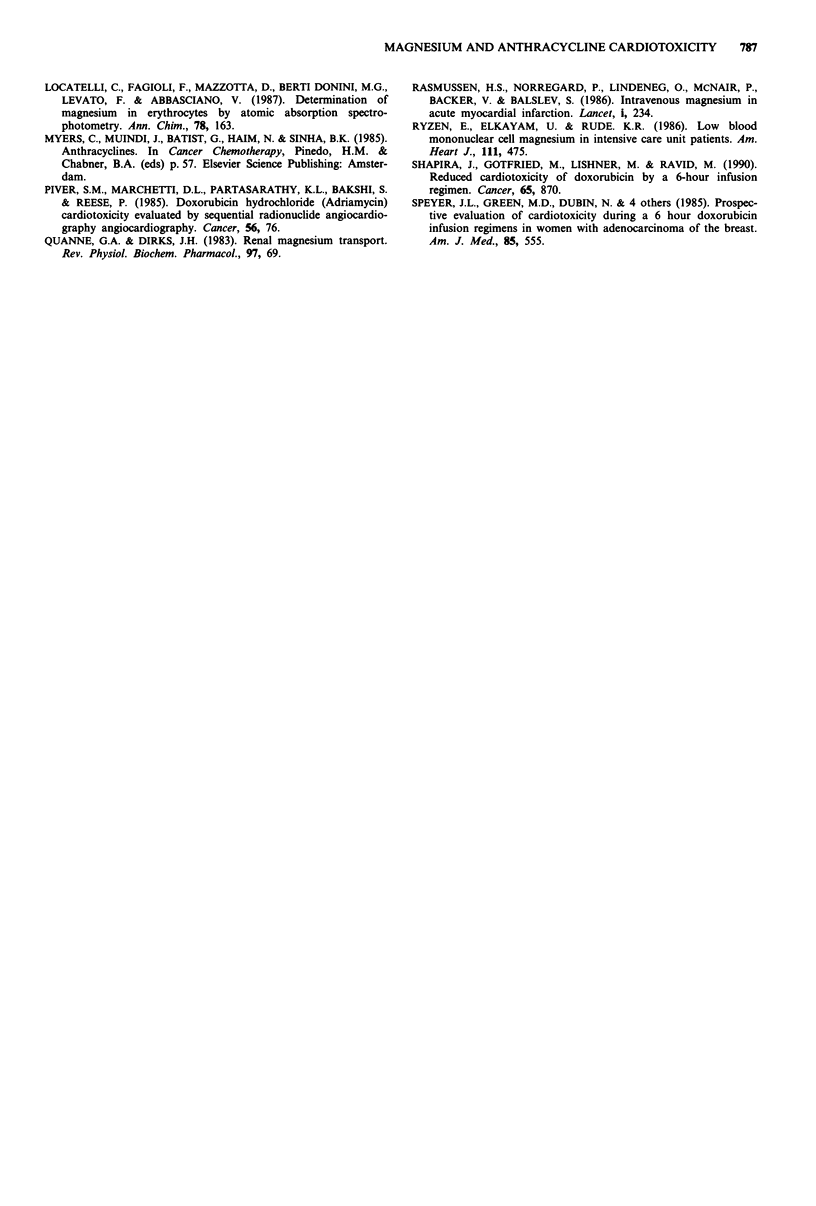# Intracellular magnesium concentrations and acute anthracycline-induced cardiotoxicity.

**DOI:** 10.1038/bjc.1991.399

**Published:** 1991-10

**Authors:** S. Sartori, I. Nielsen, D. Tassinari, A. Maestri, V. Abbasciano

**Affiliations:** II Divisione di Medicina Generale, Arcispedale S. Anna, Ferrara, Italy.


Br. J. Cancer (1991), 64, 785-787 © Macmillan Press Ltd., 1991

SHORT COMMUNICATION

### Intracellular magnesium concentrations and acute anthracycline-induced cardiotoxicity

S. Sartori[1], I. Nielsen[1], D. Tassinari[1], A. Maestri[1] & V. Abbasciano[2]

[1]*II Divisione di Medicina Generale e Servizio di Oncologia, Arcispedale S.Anna, corso Giovecca 203, 44100 Ferrara;* [2]*Istituto di Patologia Medica, Universita'di Ferrara, corso Giovecca 203, 44100 Ferrara, Italy.*

Acute and chronic cardiac toxicity is the most perplexing reaction of doxorubicin. The acute syndrome is characterised by rhythm disturbances, electrocardiographic abnormalities and occasionally acute cardiac dilatation. The chronic damage is dose-dependent and can lead to congestive heart failure. 4'-epidoxorubicin (epirubicin) has been claimed to be less cardiotoxic than doxorubicin, but the advantage is not quantitatively impressive (Chabner & Myers, 1989).

Magnesium (Mg) is essential for activation of $NA^+/K^+$ ATPase pump, which plays a major role in regulating $NA^+/K^+$ transport and in maintaining resting membrane potentials (Altura BM and Altura BT, 1984). The direct and indirect (through the effects on $K^+$ and $Ca^{++}$ metabolism) cardioprotective and antiarrhythmic properties of Mg are well known (Dyckner & Wester, 1982; Iseri, 1984). Mg deficit is associated with ventricular and supraventricular arrhythmias, symptoms of cardiac failure and electrocardiographic modifications, such low QRS voltages, extended Q-T interval and ST-T tract abnormalities (Dyckner & Wester, 1979; Dickner & Wester, 1982; Chen Wan Chun *et al.*, 1982; Rasmussen *et al.*, 1986).

Mg is mainly an intracellular cation; intracellular deficit is often not revealed by measurement of serum levels (Iseri, 1984; Ryzen *et al.*, 1986; Abbasciano *et al.*, 1988), and a reliable evaluation of effective availability of Mg is achievable only by an intracellular assay. In this study we evaluated the changes in erythrocyte Mg concentrations (EMg) during infusion of doxorubicin or epirubicin, administered alone or in combination with other antineoplastic drugs, to verify whether intracellular Mg levels might have some relationship with acute anthracycline-induced cardiotoxicity.

Twenty-six cancer patients (five males and 21 females, aged 46-63 years) gave their informed consent to the study. Tumour types and chemotherapy regimens are reported in Table I. No patient had hypertension, diabetes, coronary artery disease or electrocardiographic abnormalities, and none had received prior irradiation to the mediastinum.

EMg was measured just before (day 0) and 1, 2 and 7 days after doxorubicin or epirubicin administration. Serum levels of Mg, Na, K, Cl, Ca, CPK, LDH, ALT and AST were also measured in all patients. Urinary Mg excretion (UMg) was determined in 16 patients. EMg was assayed by atomic absorption spectrophotometry on washed and purified red blood cells, according to the method described elsewhere (Locatelli *et al.*, 1987); the other parameters were assayed using standard laboratory methods.

During the period of observation all patients received the same dietary regimen to avoid differences in Mg intake; no diuretic was administered (with the exception of intravenous furosemide 20 mg in two cases treated with the schedule cisplatin + doxorubicin + cyclophosphamide). Vomiting was controlled by intravenous metoclopramide or methylprednisolone, which do not interfere with Mg homeostasis when acutely administered (Quanne & Dirks, 1983). Each patient had daily clinical and electrocardiographic evaluation, which were done always by the same physician to ensure homogeneity in evaluating cardiac changes; the increase of 20% or more in heart rate from pretreatment values was considered as a 'minor' change; symptoms of cardiac insufficiency, rhythm disturbances and electrocardiogram (ECG) abnormalities were considered as 'major' changes. The number of vomiting episodes and the possible onset of diarrhoea was also registered for each patient. Clinical-electrocardiographic evaluation and EMg assay were done blind by two different physicians.

Essential results of the study are reported in Table I. Serum levels of Mg, Na, K, Cl, Ca, LDH, CPK, AST and ALT did not show significant changes. No symptoms of cardiac failure were observed. Four patients had increase of 20% or more in heart rate and four had 'major' changes; all cardiac disturbances were observed within 48 h from doxorubicin or epirubicin administration. EMg increased within 48 h in 15 patients and decreased in 11. Only one patient with increased EMg had 'minor' changes, whereas seven out of 11 patients (63.6%) with reduced EMg had cardiac disturbances; three out of four 'major' changes were observed in the patients who had the more marked reduction in EMg. The difference is statistically significant either considering the whole frequency of cardiac disturbances ($P < 0.01$, using Fisher's exact test) or considering the distribution in two classes of severity ($P < 0.05$, using chi-square test). The mean percentage ± Standard Deviation of change in EMg from pretreatment levels to the concentrations determined after 48 h was 10.98% ± 23.36 in the group without cardiac modifications, and − 21.85% ± 23.17 in the group with cardiac disturbances. This difference is statistically significant ($P < 0.01$ using unpaired Wilcoxon's signed-rank test). In all cases but one UMg decreased when EMg increased and vice versa. The ECG of the patients with reduced QRS voltage and ST-T tract abnormalities was still abnormal on the 7th day; in these subjects EMg remained lower than pretreatment levels. All other patients had recovered pretreatment EMg levels and had normal ECG after 7 days.

No patient had diarrhoea; there was no difference in mean number of vomiting episodes between the patients with reduced and increased EMg. No difference in EMg and UMg changes, in frequency and in severity of cardiac disturbances was observed between doxorubicin and epirubicin, nor between monochemotherapy and combination chemotherapies.

Direct measurement of myocardial Mg requires endomyocardial biopsies; this technic cannot be employed in serial studies on human subjects for obvious ethical reasons. In clinical practice, the measurement of red cell Mg is the basic method for evaluating cellular Mg metabolism (Durlach, 1988). If the changes in EMg represent in some degree similar changes in myocardial cells, our results suggest that Mg might play some role in anthracycline-induced acute

Correspondence: S. Sartori.
Received 7 February 1991; and in revised form 3 June 1991.

**Table I** Tumour types, chemotherapy regimens and essential results of the study

| *Tumour type[a] and therapy[b]* | | *EMg/UMg[c]* 0 | 1 | 2 | 7 | %[d] | Cardiac changes |
|---|---|---|---|---|---|---|---|
| 1) Ov | P + D + C | 3.36 | 4.02 | 4.12 | 4.38 | + 10.2 | None |
| 2) Br | D | 4.89/8 | 4.84/7 | 5.39/4 | 4.84/7 | + 10.2 | None |
| 3) Br | E + V | 4.71/8 | 5.82/3.8 | 5.58/5.6 | 4.84/8 | + 18.7 | None |
| 4) Br | E + V | 3.83/18 | 4.46/7.5 | 4.13/10 | 4.99/15 | + 7.8 | None |
| 5) Br | E + V | 3.98 | 3.12 | 2.32 | 2.82 | − 41.7 | Reduced QRS voltage |
| 6) Lu | V + D + C | 1.7/4.2 | 2.12/3 | 3.1/2.1 | 3.9/3.2 | + 78.1 | None |
| 7) Br | E | 3.95/9 | 4.1/4 | 4.37/6 | 4.7/5.5 | + 24 | Increased heart rate |
| 8) Br | D | 4.35/1 | 3.63/3 | 3.4/3 | 4.24/8 | − 21.8 | None |
| 9) Ov | P + D + C | 5.24/8 | 3.34/6 | 3.4/4.4 | 3.8/6.2 | − 35.1 | Reduced QRS voltage |
| 10) Br | E + V | 2.82 | 2.92 | 3.88 | 3.38 | + 37.5 | None |
| 11) Occ | D + V | 4.15 | 4.33 | 4.69 | 4.53 | + 13 | None |
| 12) Occ | D + V | 3.9/9.1 | 4.09/8 | 4.3/7.6 | 4.8/9.3 | + 9.6 | None |
| 13) Br | D | 4.84 | 3.26 | 1.82 | 3.87 | − 62.4 | Tachyfibrillation (170/min) |
| 14) Lu | V + D + C | 3.06/7 | 3.4/6.1 | 2.73/8 | 3.04/9 | − 10.8 | Increased heart rate |
| 15) Br | D + V | 3.7/4 | 3.2/6 | 3.5/7 | 3.7/3 | − 5.4 | Increased heart rate |
| 16) Br | E | 2.84 | 2.91 | 3.34 | 3.28 | + 17.6 | None |
| 17) Lu | V + D + C | 4.12/7.4 | 4.72/6 | 4.51/5.6 | 4.34/7 | + 9.4 | None |
| 18) Br | E | 3.75 | 4.02 | 4.05 | 4.02 | + 8 | None |
| 19) Occ | E + V | 2.26 | 2.38 | 2.63 | 2.51 | + 16.3 | None |
| 20) Br | D | 4.48/8.4 | 4.27/8.4 | 3.83/11 | 4.3/10.8 | − 14.5 | None |
| 21) Br | E + V | 5.38/5 | 5.02/6.7 | 4.6/7.5 | 4.47/10 | − 16.9 | ST-T flattening and inversion |
| 22) Occ | D + V | 4.03/6 | 4.2/7 | 3.27/8.5 | 4.1/4.2 | − 18.8 | None |
| 23) Br | D + V | 3.39 | 4.14 | 4.09 | 3.48 | + 20.6 | None |
| 24) Br | E + V | 3.52 | 3.52 | 3.68 | 3.61 | + 4.5 | None |
| 25) Occ | E | 4.21/7 | 3.94/9 | 3.32/9.9 | 4.28/8 | − 21.1 | None |
| 26) Br | E + V | 3.28/5.2 | 2.8/7.4 | 2.85/9 | 3.3/8.2 | − 13.1 | Increased heart rate |

[a]Br = Breast cancer; Lu = Lung cancer; Ov = Ovarian cancer; Occ = Occult neoplasia. [b]D = Doxorubicin 50 mg mq$^{-1}$ body surface area; E = Epirubicin 75 mg mq$^{-1}$ b.s.a.; P = Cisplatin 50 mg mq$^{-1}$ b.s.a.; V = Vincristine 1.2 mg mq$^{-1}$ b.s.a.; C = Cyclophosphamide 500 mg mq$^{-1}$ b.s.a. in ovarian cancer and 750 mg mq$^{-1}$ b.s.a. in lung cancer. [c]EMg = Erythrocyte Mg concentration (in Meq/l) UMg = Urinary Mg excretion (in Meq/24 h) measured before (day 0) and 1, 2 and 7 days after chemotherapy administration. [d]Percentage of change in EMg from day 0 to day 2.

cardiotoxicity. Most of the existing evidence supports free-radicals formation as the basis for acute cardiotoxicity induced by anthracyclines; moreover doxorubicin reduces glutathione peroxidase activity, a key enzyme in the detoxication of peroxides (Myers *et al.*, 1985; Chabner & Myers, 1989). Anthracyclines also have a direct action on cell membrane, causing fluidity changes and alterating the organisation of membrane phospholipids (Myers *et al.*, 1985). Mg has a stabilising effect for the cell membrane; its complexing with phospholipids reduces membrane fluidity and permeability (Durlach, 1988). Moreover Mg is pivotal in the synthesis of glutathione and in the transfer, storage and utilisation of energy, regulates redox reactions and maintains the coupling of oxidation and phosphorylation in mitochondria (Iseri, 1984; Durlach, 1988). All these functions could be useful in protecting myocardial cells against anthracycline-induced injury, and it is possible that the cell requirement of Mg increases during the administration of doxorubicin and epirubicin, to counterbalance the toxic effects of the drugs. In some predisposed patients the cells might be unable to increase their Mg content, owing to enhanced susceptibility to the direct action of anthracyclines on cell membrane, with formation of covalent bindings to membrane structures (Myers *et al.*, 1985) and consequent leakage of membrane Mg and/or inability of the cells to uptake serum Mg. The risk of acute cardiotoxicity would be higher in such patients: indeed we obserged 'major' ECG changes only in patients with reduced EMg.

Prolonged infusion of doxorubicin seems to reduce chronic cardiotoxicity (Speyer *et al.*, 1985). This observation supports the hypothesis that cardiotoxicity is related to repeated high concentrations of the drug, which would induce an acute myocarditis-like effect (Shapira *et al.*, 1990), rather than to the total cumulative dose (Alexander *et al.*, 1979): the sum of repeated acute toxic effects would lead to the chronic damage (Piver *et al.*, 1985). Our preliminary data suggest a relationship between intracellular Mg concentrations and anthracycline-induced acute toxic effects on the heart: might the monitoring of intracellular Mg levels have some value in predicting the development of the chronic damage? More work is necessary to test this idea.

This research was supported by Italian 'Ministero dell'Universita' e della Ricerca Scientifica e Tecnologica'. The authors acknowledge the technical assistance of LIRCA s.p.a.

### References

ABBASCIANO, V., LEVATO, F., REALI, M.G. & 5 others (1988). Reduction of erythrocyte magnesium concentration in heterozygote beta-thalassemic subjects and in normal subjects submitted to physical stress. *Mag. Res.*, **1**, 213.

ALEXANDER, J., DAINIAK, N., BERGER, J.Y. & 4 others (1979). Serial assessment of doxorubicin cardiotoxicity with quantitative radionuclide angiocardiography. *N. Engl. J. Med.*, **300**, 278.

ALTURA, B.M. & ALTURA, B.T. (1984). Magnesium, electrolyte transport and coronary vascular tone. *Drugs*, **28** (Suppl. 1), 120.

CHABNER, B.A. & MYERS, C.E. (1989). Clinical pharmacology of cancer chemotherapy. In *Cancer, Principles and Practice of Oncology*, De Vita, V.T., Hellman, S. & Rosemberg, S.A. (eds) p. 349, Lippincott: Philadelphia.

CHEN WAN-CHUN, FU XIN-XIANG, PAN ZHEN-JIA & QIAN SHAO-ZEN (1982). ECG changes in early stage of magnesium deficiency. *Am. Heart J.*, **104**, 1115.

DYCKNER, T. & WESTER, P.O. (1979). Ventricular extrasystoles and intracellular electrolytes before and after potassium and magnesium infusions in patients on diuretic treatment. *Am. Heart J.*, **97**, 12.

DYCKNER, T. & WESTER, P.O. (1982). Magnesium in cardiology. *Acta Med. Scand.*, **611** (Suppl), 27.

DURLACH, J. (1988). *Magnesium in Clinical Practice*. John Libbey & Co: London.

ISERI, L.T. (1984). Magnesium in coronary artery disease. *Drugs*, **28** (Suppl 1), 151.

LOCATELLI, C., FAGIOLI, F., MAZZOTTA, D., BERTI DONINI, M.G., LEVATO, F. & ABBASCIANO, V. (1987). Determination of magnesium in erythrocytes by atomic absorption spectrophotometry. *Ann. Chim.*, **78**, 163.

MYERS, C., MUINDI, J., BATIST, G., HAIM, N. & SINHA, B.K. (1985). Anthracyclines. In *Cancer Chemotherapy*, Pinedo, H.M. & Chabner, B.A. (eds) p. 57. Elsevier Science Publishing: Amsterdam.

PIVER, S.M., MARCHETTI, D.L., PARTASARATHY, K.L., BAKSHI, S. & REESE, P. (1985). Doxorubicin hydrochloride (Adriamycin) cardiotoxicity evaluated by sequential radionuclide angiocardiography angiocardiography. *Cancer*, **56**, 76.

QUANNE, G.A. & DIRKS, J.H. (1983). Renal magnesium transport. *Rev. Physiol. Biochem. Pharmacol.*, **97**, 69.

RASMUSSEN, H.S., NORREGARD, P., LINDENEG, O., MCNAIR, P., BACKER, V. & BALSLEV, S. (1986). Intravenous magnesium in acute myocardial infarction. *Lancet*, **i**, 234.

RYZEN, E., ELKAYAM, U. & RUDE. K.R. (1986). Low blood mononuclear cell magnesium in intensive care unit patients. *Am. Heart J.*, **111**, 475.

SHAPIRA, J., GOTFRIED, M., LISHNER, M. & RAVID, M. (1990). Reduced cardiotoxicity of doxorubicin by a 6-hour infusion regimen. *Cancer*, **65**, 870.

SPEYER, J.L., GREEN, M.D., DUBIN, N. & 4 others (1985). Prospective evaluation of cardiotoxicity during a 6 hour doxorubicin infusion regimens in women with adenocarcinoma of the breast. *Am. J. Med.*, **85**, 555.